# CLINICAL, DEMOGRAPHIC, ANATOMOPATHOLOGICAL, AND MOLECULAR FINDINGS IN PATIENTS WITH MEDULLOBLASTOMA TREATED IN A SINGLE HEALTH FACILITY

**DOI:** 10.1590/1984-0462/2021/39/2019298

**Published:** 2020-11-16

**Authors:** Iva Loureiro Hoffmann, Izilda Aparecida Cardinalli, José Andrés Yunes, Ana Luiza Seidinger, Ricardo Mendes Pereira

**Affiliations:** a Centro Infantil de Investigações Hematológicas Dr. Domingos A. Boldrini, Campinas, SP, Brazil.; b Universidade Estadual de Campinas, Campinas, SP, Brazil.

**Keywords:** Medulloblastoma, Central nervous system neoplasms, Medical oncology, Meduloblastoma, Neoplasias do sistema nervoso central, Oncologia

## Abstract

**Objective:**

To describe the clinical, demographic, anatomopathological, molecular, and survival characteristics of patients with medulloblastoma.

**Methods:**

Retrospective study based on patient information obtained from the review of medical records. Overall and event-free survival were analyzed using the Kaplan-Meier estimator, and the curves were compared by the log-rank test.

**Results:**

Among the patients investigated, 70 were male (66%), and age at diagnosis ranged from 2 months to 22 years. The most frequent signs and symptoms were headache (80.8%) and vomiting (75.8%). Regarding treatment, most patients (63.2%) underwent complete surgical resection, with a predominance of classic histology (63.2%). The 5-year overall survival rate was 67.9%, and the 10-year rate was 64.2%. Patients with molecular profile characteristic of the wingless (WNT) subgroup had a better prognosis, with 5-year overall survival of 75%.

**Conclusions:**

The clinical, demographic, anatomopathological, and molecular characteristics of patients with medulloblastoma described in the present study were mostly similar to those reported in the literature. Patients submitted to complete tumor resection had better clinical outcomes than those who underwent incomplete resection/biopsy. Patients classified as high-risk showed worse overall and event-free survival than those in the standard-risk group, and the presence of metastasis at diagnosis was associated with recurrence.

## RESUMO

**Objetivo:**

Descrever as características clínicas, demográficas, anatomopatológicas, moleculares e de sobrevida de pacientes portadores de meduloblastoma.

**Métodos:**

Estudo retrospectivo, no qual as informações dos pacientes foram obtidas pela revisão dos prontuários médicos. Análises de sobrevida global e de sobrevida livre de eventos foram realizadas por meio da construção de curvas de Kaplan-Meier e a comparação entre as curvas foi feita pelo teste *log-rank.*

**Resultados:**

Entre os pacientes analisados, 70 pertenciam ao sexo masculino (66%) e a idade ao diagnóstico variou de dois meses a 22 anos. Os sinais e sintomas de maior frequência foram cefaleia (80,8%) e vômitos (75,8%). Em relação ao tratamento, a maioria (63,2%) dos pacientes foi submetida à ressecção cirúrgica total e apresentava como histologia predominante a forma clássica (63,2%). A taxa de sobrevida global em cinco anos foi de 67,9% e, em 10 anos, de 64,2%. Os pacientes com perfil molecular característico do subgrupo *wingless* (WNT) apresentaram melhor prognóstico, com sobrevida global em cinco anos de 75%.

**Conclusões:**

As características clínicas, demográficas, anatomopatológicas e moleculares dos pacientes com meduloblastoma descritas no presente estudo foram majoritariamente semelhantes às descritas na literatura. Pacientes submetidos à ressecção completa do tumor tiveram melhor evolução clínica do que aqueles com ressecção incompleta/biópsia. Pacientes estratificados como de alto risco apresentaram pior sobrevida global e livre de eventos do que o grupo *standard* e a presença de metástases ao diagnóstico se mostrou associada à ocorrência de recidiva da doença.

## INTRODUCTION

Central nervous system (CNS) neoplasms are the second most frequent type of cancer reported in children and adolescents.^1^ ^,^ ^2^ CNS tumors represent the main cause of cancer-related mortality. In addition, the sequelae caused by tumors and their treatments result in high morbidity among children.^3^ ^,^ ^4^ ^,^ ^5^

Medulloblastoma, a primitive neuroectodermal tumor originated in the cerebellum, is one of the most common malignant brain tumors in children, representing 20% of CNS tumors. It is an invasive tumor, classified as grade IV by the World Health Organization (WHO),^6^ whose prognosis is particularly unfavorable to children under three years of age. In adults, the incidence is low, ranging from 0.5 to 1%.^7^

Patients diagnosed with medulloblastoma are stratified according to age, extension of the tumor resection, and presence of metastasis. Patients older than three years with completely resected tumors and without metastasis at diagnosis are classified as standard-risk, while all other cases are considered high-risk. Based on this stratification and with the use of modern multimodal therapy, approximately 70% of children with medulloblastoma survive until adulthood.^8^ ^,^ ^9^ ^,^ ^10^ Despite the relative improvement in patient survival, the endocrine side effects and neurocognitive sequelae resulting from therapy, particularly in children under seven years of age, represent a challenge in the treatment of medulloblastoma.^5^

In 2016, WHO updated the classification of CNS tumors and included the following medulloblastoma molecular subgroups: wingless (WNT) tumors, sonic hedgehog (SHH) tumors without *TP53* mutation, SHH tumors with *TP53* mutation, and non-WNT/non-SHH tumors.^11^ The medulloblastoma molecular classification should help define the risk stratification in future therapeutic protocols. Patients with good prognosis (WNT subtype) should be considered for protocols with reduced therapy, while those with poor prognosis (non-WNT/non-SHH subtype) should be prioritized in experimental therapies.^12^

The introduction of the new WHO classification, as well as the stratification and new therapeutic protocols based on molecular data, will enable devising more effective approaches to fill the gaps of the medulloblastoma treatment. To that end, we must evaluate the current results of patients treated in a Brazilian center specialized in pediatric hematology-oncology. The present study aims to describe the clinical, demographic, anatomopathological, molecular, and survival characteristics of patients with medulloblastoma.

## METHOD

In the study period (between January 2002 and December 2017), 4,519 new cancer patients were treated in a single health facility specialized in pediatric hematology-oncology located in Campinas, São Paulo. Among them, 712 (15%) had CNS tumors, of whom 120 (2.7% of the total; 17% of CNS tumors) were diagnosed with medulloblastoma. Out of the 120 patients with medulloblastoma, 14 (11.7%) were excluded from the analysis - 4 due to loss to follow-up, and 10 because they received treatment in other facilities.

This is a retrospective cohort study based on patient information obtained from the review of medical records. We collected the following data: date of birth, gender, age at diagnosis, date of diagnosis, symptoms, interval between the onset of signs and symptoms and diagnosis, surgery, histology, molecular profile, staging, metastasis, radiotherapy, chemotherapy, image data, date of the last visit, current clinical status, recurrence, sequelae, and end of therapy.

Patient age was categorized into three clinically significant groups: children under 3 years (<3); children aged ≥3 years and ≤16 years; young people and adults over 16 years (>16). Age and presence or absence of metastasis were evaluated at the time of diagnosis.

Tumors were histologically classified as: classic, desmoplastic, and anaplastic/large cells (A/LC).^6^ The degree of surgical resection was divided into three groups: total (complete resection or less than 1.5 cm^2^ of residual tumor), partial (1.5 cm^2^ or more of residual tumor), and biopsy only. The quantitative analysis of residual tumor was performed by magnetic resonance imaging (MRI) or computed tomography (CT) of the brain after surgery.

The cases were stratified into two risk groups:

- “standard-risk”: patients ≥3 years of age, with completely resected tumors and no metastatic disease.
- “high-risk”: the remaining cases.

In cases with molecular evaluation, the tumors were categorized into three subgroups, according to the WHO criterion (2016): WNT, SHH, and non-WNT/non-SHH.^11^ Molecular subtypes were determined by quantitative polymerase chain reaction (PCR) for the expression analysis of differentially expressed genes in each group.

In the statistical analysis, we calculated frequencies and percentages of qualitative variables. We tested the association between qualitative variables using Fisher’s exact test. For the analysis of overall survival, the initial date was defined as that of diagnosis and the final date as that of death or the latest information obtained in evaluation in January 2019. For the analysis of event-free survival, the initial date was established as that of diagnosis and the final date as that of recurrence or death, whichever occurred first. Recurrence was defined as the reappearance of the tumor lesion in the imaging test in the site of the original tumor or any other site or the presence of neoplastic cells in the cerebrospinal fluid. Overall and event-free survival were analyzed using the Kaplan-Meier estimator, and the curves were compared by the log-rank test. For all analyses, we adopted a 5% significance level and used the IBM SPSS Statistics software, version 25.

The Research Ethics Committee of Centro Infantil Boldrini (CIB) approved this research, under the Certificate of Presentation for Ethical Consideration (*Certificado de Apresentação para Apreciação Ética* - CAAE) no. 51995415.5.5.0000.5376. Guardians of patients who were still on follow-up signed an informed consent form, and we asked the Research Ethics Committee to waive the form for those who had died or were no longer being monitored at the unit, which was granted.

## RESULTS

Among the 106 patients analyzed, 70 (66%) were male, and 36 (34%) were female, resulting in a male:female ratio of 1.94:1. Age at diagnosis ranged from 2 months and 2 days to 22 years and 1 month, with an average of 7 years and 4 months, and a median of 7 years. The interval between the onset of symptoms and diagnosis ranged from 3 days to 3 years, with a mean of 3.2 months and a median of 2 months. The most frequent signs and symptoms were vomiting (80.8%), headache (75.8%), and motor impairment (58.3%).

Throughout the study period, patients received the same treatment protocol. Those with standard risk received chemotherapy and radiotherapy in the brain and spine of 23.4 Gy, with a boost to the posterior fossa of 30.6 Gy. High-risk patients aged three years or older received chemotherapy and radiotherapy in the neuraxis of 36 Gy, with a boost to the posterior fossa of 18 Gy, while those under three years underwent only chemotherapy. All chemotherapy was based on protocols and used the following drugs: cisplatin/carboplatin, vincristine, ifosfamide/cyclophosphamide, and etoposide.

Twenty-four patients presented no treatment-related sequelae; the others had at least one sequela, usually motor impairment/ataxia and endocrine changes. We found no second neoplasms. Table 1 shows the general characteristics of the patients.

**Table 1:** Clinical and demographic characteristics of pediatric patients with medulloblastoma treated in a Brazilian reference center (n=106), Campinas, São Paulo.

	Total=106	
	n	%
Age (years)		
<3	15	14.1
3-16	87	82.1
>16	04	3.8
Tumor site in the radiological examination		
4^th^ ventricle	73	68.9
Cerebellar hemisphere	22	20.8
Middle cerebellar peduncle	10	9.4
No information	01	0.9
Histological diagnosis		
Classic	67	63.2
Desmoplastic	29	27.3
Anaplastic/large cells	10	9.5
Clinical staging		
High-risk	64	60.4
Standard-risk	42	39.6
Surgery		
Complete resection	67	63.2
Incomplete resection	36	34.0
Biopsy	03	2.8
Radiotherapy		
Skull and neuraxis	88	83.0
Not performed	18	17.0
Recurrence		
Yes	28	26.4
No	78	73.6
Status at the last follow-up (January 2019)		
Alive without disease	66	62.3
Alive with disease	01	0.9
Died of disease	27	25.5
Died of infection	07	6.6
Died of surgical complications	05	4.7

The frequency of males and females among patients aged three years or older resulted in a ratio of 1.67:1. However, among patients younger than three years, the ratio was 6.5:1 (p=0.117). The classic presentation predominated in the age group 3 to 16 years, and the desmoplastic was more frequent in patients under 3 years (p=0.021). None of the 4 patients older than 16 years had metastases, while 4/15 of those under 3 years and 38/87 aged from 3 to 16 years were metastatic (p=0.109). We identified no statistical association between the incidence of recurrence and age group (p=1).

**Figure 1:**
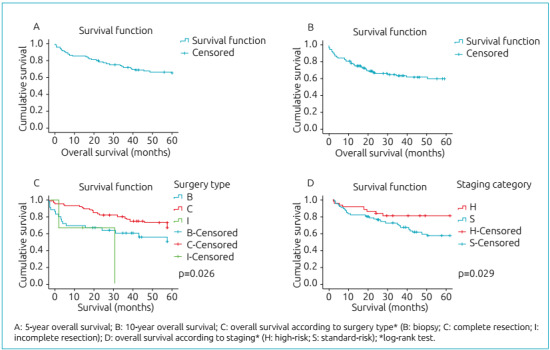
Survival curves of patients with medulloblastoma included in the study (n=106).

The patients included in the study were followed for an average of 45.9 months, ranging from 2 days to 238 months. The 5-year overall survival rate was 67.9% (Figure 1A), and the 10-year rate was 64.2% (Figure 1B). Figure 1C represents the comparison of overall survival according to the type of surgery undergone by the patients. Figure 1D shows the overall survival according to the risk group. Patients classified as high-risk and those submitted only to tumor biopsy had a more unfavorable 5-year overall survival compared to other groups (Figures 1C and 1D). The 5-year event-free survival was 60.4%, and the 10-year rate was 57.5%. Table 2 describes the association of demographic and clinical variables with the overall and event-free survival.

**Table 2:** Analysis of 5-year overall and event-free survival according to different demographic, histological, and clinical variables of pediatric patients with medulloblastoma treated in a Brazilian reference center (n=106), Campinas, São Paulo.

	Total cases	5-year overall survival rate (%)	p-value*	5-year event-free survival rate (%)	p-value*
Age group (years)					
<3	15	60.0	0.731	53.3	0.663
3-16	87	69.0		62.1	
>16	4	75.0		50.0	
Gender					
Female	36	75.0	0.395	66.7	0.533
Male	70	64.3		57.1	
Surgery					
Complete	67	68.7	**0.026**	61.2	0.12
Incomplete	36	55.6		52.8	
Biopsy	3	33.3		33.3	
Histology					
Classic	67	61.2	0.070	53.7	0.128
Desmoplastic	29	86.2		75.9	
A/LC	10	60.0		60.0	
Metastasis					
Present	42	59.5	0.183	52.4	0.231
Absent	64	73.4		65.6	
Risk					
High	64	59.4	**0.029**	51.6	**0.028**
Standard	42	81.0		3.8	

*Log-rank test; A/LC: anaplastic/large cells.

**Figure 2:**
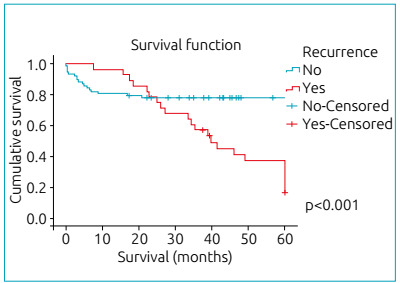
Overall survival curve of patients with medulloblastoma according to the presence (yes; n=28) or absence (no; n=78) of recurrence.

Among the 106 patients analyzed, 28 (26.4%) presented recurrence, of whom 9 belonged to the standard group. Out of the 28 cases with recurrence, 22 progressed to death from disease, 1 patient was alive with disease, and 5 were alive without disease, resulting in 5-year overall survival of 21.4%. Figure 2 illustrates the comparison of the 5-year overall survival of patients with and without recurrence (p<0.001). The presence of metastasis at diagnosis was statistically associated with the incidence of recurrence in the studied group (p=0.049).

Only 49 of the 106 cases of medulloblastoma analyzed had the molecular profile specified. The non-WNT/non-SHH subgroup was the largest, with 49% (24/49) of cases, followed by the SHH subgroup with 34.7% (17/49). The WNT subgroup comprised 16.3% (8/49) of cases and had the lowest number of patients. Regarding the risk stratification, 75% of non-WNT/non-SHH patients and 68.8% of SHH cases were considered high-risk, while 62.5% of the WNT subgroup were classified as standard-risk. Table 3 presents the association of molecular data with demographic and clinical variables, as well as the analyses of overall and event-free survival.

**Table 3:** Association between molecular subtypes and demographic, histological, and metastasis at diagnosis data from 49 patients and analysis of 5-year overall and event-free survival.

	WNT	SHH	Non-WNT/non-SHH	p-value*
Gender				
Female	5	4	7	0.134
Male	3	13	17	
Age (years)				
<3	0	7	1	**0.005**
3-16	8	10	23	
>16	0	0	0	
Histology				
Classic	5	5	19	**0.008**
Desmoplastic	2	11	4	
A/LC	1	1	1	
Metastasis				
Present	2	4	17	**0.005**
Absent	6	13	7	
Overall survival** (%)	75	58.8	66.7	0.762
Event-free survival**(%)	75	52.9	41.7	0.547

*Fisher’s exact test; **Log-rank test; WNT: wingless; SHH: sonic hedgehog; A/LC: anaplastic/large cells.

Seven of the 49 patients showed *TP53* gene mutation. The presence of mutations in this gene was not associated with patient prognosis during the study follow-up period. The 5-year overall survival in the group with *TP53* mutation was 57% versus 61% in the group without mutation (p=0.408).

## DISCUSSION

The present study analyzed 106 cases of medulloblastoma treated in a single health facility specialized in pediatric hematology-oncology in the city of Campinas, São Paulo. The clinical, demographic, anatomopathological, and molecular characteristics of patients with medulloblastoma described in the present study were mostly similar to those reported in the literature.

The frequency of medulloblastoma in the sample analyzed was higher in children aged 3 to 16 years (87 cases), with a peak between 7 and 9 years. Among the 106 cases, 96.2% occurred before 16 years of age, a frequency higher than that described in WHO studies.^6^ ^,^ ^13^

The ratio between males and females was 1.94:1, agreeing with demographic findings from other series of pediatric patients with medulloblastoma and with WHO data.^6^ ^,^ ^14^ ^,^ ^15^ However, among patients younger than 3 years, the ratio was 6.5:1. This difference was not significant when compared to the gender distribution in other age groups.

The postoperative presence of residual tumor is an important prognostic factor.^8^ In our study, the group submitted to complete tumor resection had better survival, corroborating data reported in the literature. This finding highlights the importance of safe and specialized surgical resection for the clinical outcome of cancer patients.

The WHO brain tumor classification divides medulloblastoma based on histopathological criteria - classic (more frequent), desmoplastic, and A/LC.^11^ ^,^ ^13^ ^,^ ^16^ ^,^ ^17^ ^,^ ^18^ In the sample evaluated, the classic tumor was the most frequent (63.2%), followed by the desmoplastic (27.3%) and the A/LC (9.5%). Compared to most published studies, the frequency of desmoplastic and A/LC tumors was higher in the present research, as the frequencies reported in the literature are 20 and 5%, respectively.^19^

Literature data indicate that patients with desmoplastic tumors have a better prognosis, whereas those with A/LC present a poor one.^17^ ^,^ ^20^ In this study, patients with desmoplastic tumor showed the best overall survival (86.2%), while those with A/LC had the worst (60%). Although these differences are important, we found no statistical significance. Among children younger than three years, there was only one A/LC case. Most desmoplastic cases were detected in patients under 16 years of age, corroborating previous assumptions that desmoplastic medulloblastomas are more common in infants and younger children.^21^

Children under three years of age had the worst overall survival (60%). This finding can be explained by the fact that children from this age group are considered “high-risk,” and this risk group showed a poorer prognosis compared to standard-risk patients. The immature CNS of these children prevents them from receiving the same dose of radiotherapy offered to older patients due to the greater risk of neurological, endocrine, and psychosocial sequelae. Together, these data suggest that the treatment proposed for children under three years of age may be disproportional to the aggressiveness of the neoplasm, which would explain the low survival.^17^

Each component of the treatment can cause late complications that might have a profound effect on the quality of life and longevity of medulloblastoma survivors.^18^ Although the late effects of treatment on the quality of life are often attributed to craniospinal irradiation, chemotherapy has a significant role in the worsening of radiotherapy adverse effects.^22^ ^,^ ^23^ The main late effects are: neurocognitive impairment, motor abnormalities, endocrine changes, and second malignancies. Similar sequelae were found in our study, except for second neoplasms.

One-third of medulloblastomas can metastasize to any CNS region by following the cerebrospinal fluid canals.^8^ ^,^ ^24^ In our research, 42 (39.63%) patients had metastasis at diagnosis, and, as expected, the presence of metastasis was associated with a tendency to a worse clinical course, given that the 5-year survival rate in this group was 59.5%, while in those without metastasis, the rate was 73.4%, even though we did not find statistical significance. The presence/absence of metastasis at diagnosis was an important factor associated with the incidence of recurrence.

The current risk stratification model is based on variables such as age, extension of tumor resection, and presence of metastasis, and the latter showed a significant correlation with overall and event-free survival in patients with medulloblastoma. However, we underline that 21% (9/42) of patients classified as standard-risk had recurrence. This finding suggests that the current stratification model does not adequately predict the variability of prognosis in patients allocated in each group. Many children classified as standard-risk and with favorable prognosis may currently be receiving excessive treatment. That is the case of patients with WNT tumors, which present good clinical outcomes and rarely metastasize. On the other hand, patients from this group may be undertreated. For this reason, using the medulloblastoma molecular classification is essential for guiding the risk stratification of future therapy protocols. Patients with good prognosis (WNT subtype) should be considered for protocols with reduced therapy, while those with poor prognosis (non-WNT/non-SHH subtype) should be prioritized in experimental therapies.^12^

Out of the 49 cases whose medical reports included the molecular subtype, 8 were classified as WNT (16.3%), 17 as SHH (34.7%), and 24 as non-WNT/non-SHH (49%). The absolute frequencies found in the subgroups differed from those described in the literature; however, the proportional distribution between cases was similar, with non-WNT/non-SHH as the most frequent, followed by SHH and WNT.^15^ ^,^ ^25^

The data obtained showed that the demographic and clinical profile of the WNT subgroup is very similar to that identified in other series of patients, which presented a good prognosis and male:female ratio of 1:1. The SHH subgroup had an incidence of 34.7% in the current study, while the literature reports 28%. Also, the male:female ratio (3.2:1) in the SHH subgroup was higher when compared to other studies.^15^ ^,^ ^25^ ^,^ ^26^ These differences can be explained by the relatively small sample; therefore, increasing the number of patients classified according to their molecular profile is important. For comparison purposes, we analyzed the results of the present study together with those presented in an international meta-analysis that included 550 patients from 7 independent studies^14^ and with the current consensus on medulloblastoma molecular subgroups.^25^ Table 4 shows a comparative overview of these studies regarding the frequencies estimated for each subtype, as well as the demographic, histological, and clinical variables described for each of them.

**Table 4:** Comparative analysis between the results of the present study and those reported in the literature for each medulloblastoma subtype.

	Present study data	Literature data
Frequency		
WNT	16.3%	11.0%^14^
SHH	34.7%	28.0%^14^
Non-WNT/non-SHH group	49.0%	61.0%^14^
Gender (M:F)		
WNT	0.6:1	1:1^14^ ^,^ ^25^
SHH	3.2:1	1:1^14^ ^,^ ^25^
Non-WNT/non-SHH group	2.4:1	2:1^14^ ^,^ ^25^
Age group		
WNT	All 3-16	Mostly 3-16^14^ ^,^ ^25^
SHH	Mostly 3-16	Mostly <3 or >16^14^ ^,^ ^25^
Non-WNT/non-SHH group	Mostly 3-16	<3 and 3-16^14^ ^,^ ^25^
Histology		
WNT	Mostly classic	Mostly classic^14^ ^,^ ^25^
SHH	Mostly desmoplastic	Classic, desmoplastic, A/LC^14^ ^,^ ^25^
Non-WNT/non-SHH group	Mostly classic	Classic, A/LC^14^ ^,^ ^25^
Metastasis		
WNT	25%	Rare (9%)^14^ ^,^ ^25^
SHH	23%	Unusual (17-22%)^14^ ^,^ ^25^
Non-WNT/non-SHH group	70%	Very frequent (30-47%)^14^ ^,^ ^25^
Prognosis*		
WNT	75%	Better (>90%)^14^ ^,^ ^25^
SHH	58.8%	Intermediate (68-75%)^14^ ^,^ ^25^
Non-WNT/non-SHH group	66.7%	Worse (45-58%)^14^ ^,^ ^25^

*Five-year survival; WNT: wingless; SHH: sonic hedgehog; M: male; F: female; A/LC: anaplastic/large cells.

*TP53* gene mutations are reported in approximately 10% of patients with medulloblastoma.^14^ In the present study, 14% of patients showed mutations in this gene (n=7/49). The presence of these mutations was not associated with a tendency to a worse clinical course, contrary to reports from other series of patients in the literature. Nonetheless, we emphasize that the number of patients with mutation was small (n=7); therefore, increasing the number of cases evaluated would be necessary to obtain more conclusive data.

As medulloblastoma is a relatively rare disease, the study of a cohort with 106 patients treated in a single health facility is very relevant, but insufficient for certain comparative analyses, demonstrating the need for multicenter studies. The clinical, demographic, anatomopathological, and molecular characteristics of pediatric patients with medulloblastoma described in the present study were mostly similar to those reported in the literature. This information and the continuous search for validation of global evidence with respect to local problems enables the participation of Brazilian facilities in international multicenter protocols for the treatment of medulloblastoma, which can significantly improve the clinical outcomes achieved.^27^

The systematic knowledge of the molecular biology of medulloblastoma is crucial because it will allow the emergence of new specific therapeutic modalities focused on molecular targets, aiming at increasing survival and reducing treatment-related morbidities.
