# ERRATUM

**DOI:** 10.1590/1984-0462/2021/39/2019298erratum

**Published:** 2020-12-21

In the manuscript “Clinical, demographic, anatomopathological, and molecular findings in patients with medulloblastoma treated in a single health facility”, DOI: http://dx.doi.org/10.1590/1984-0462/2021/39/2019298, published in the Rev. Paul. pediatr. [Internet]. 2020;39:e2019298. Epub November 11, 2020, in page 4.

Where it reads:

**Figure 1:**
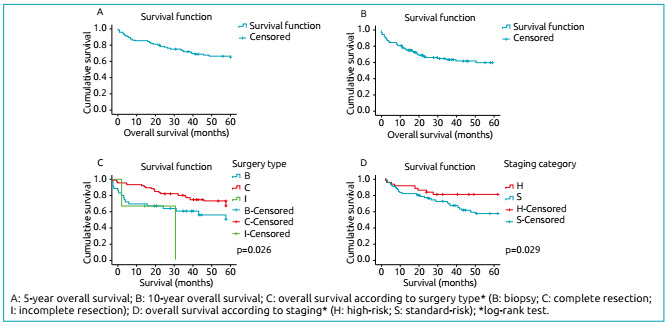
Survival curves of patients with medulloblastoma included in the study (n=106).

It should read:

**Figure 1:**
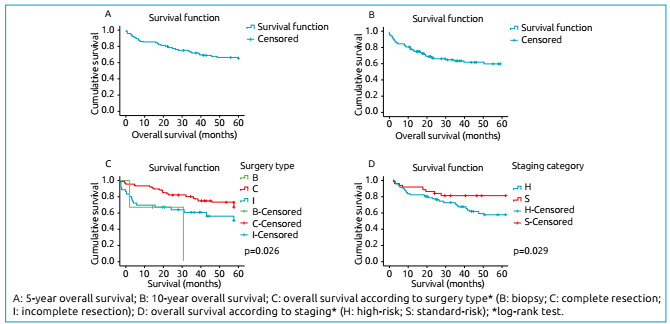
Survival curves of patients with medulloblastoma included in the study (n=106).

